# COVID-19 Serological Tests: How Well Do They Actually Perform?

**DOI:** 10.3390/diagnostics10070453

**Published:** 2020-07-04

**Authors:** Abdi Ghaffari, Robyn Meurant, Ali Ardakani

**Affiliations:** 1 Novateur Ventures Inc., Vancouver, BC V6E 3P3, Canada; 2 Department of Pathology and Molecular Medicine, Queen’s University, Kingston, ON K7L 3N6, Canada; 3 NSF Health Sciences, Kirkbymoorside, York YO62 6AF, UK; rmeurant@nsf.org

**Keywords:** COVID-19, serological diagnostic test, SARS-CoV-2, performance

## Abstract

In only a few months after initial discovery in Wuhan, China, SARS-CoV-2 and the associated coronavirus disease 2019 (COVID-19) have become a global pandemic causing significant mortality and morbidity and implementation of strict isolation measures. In the absence of vaccines and effective therapeutics, reliable serological testing must be a key element of public health policy to control further spread of the disease and gradually remove quarantine measures. Serological diagnostic tests are being increasingly used to provide a broader understanding of COVID-19 incidence and to assess immunity status in the population. However, there are discrepancies between claimed and actual performance data for serological diagnostic tests on the market. In this study, we conducted a review of independent studies evaluating the performance of SARS-CoV-2 serological tests. We found significant variability in the accuracy of marketed tests and highlight several lab-based and point-of-care rapid serological tests with high levels of performance. The findings of this review highlight the need for ongoing independent evaluations of commercialized COVID-19 diagnostic tests.

## 1. Background

Coronavirus disease 2019 (COVID-19) was first discovered in a cluster of patients with severe respiratory symptoms in Hubei Province, China, in December 2019. The early nucleic acid analysis of known pathogen panels led to negative results, suggesting the causative agent was of unknown origin. By early January 2020, analysis of bronchoalveolar lavage (BAL) fluid from infected patients revealed a pathogen, later named SARS-CoV-2, with 50%, 80%, and 96% genetic sequence overlap to the genome of the Middle East respiratory syndrome virus (MERS-CoV), the severe acute respiratory syndrome virus (SARS-CoV), and bat coronavirus RaTG13, respectively [1,2]. Like SARS-CoV and MERS-CoV, SARS-CoV-2 is a single-stranded RNA virus belonging to the beta genus *Coronavirus* in the *Coronaviridae* family [3].

As SARS-CoV-2 can be transmitted from human to human, the disease has spread swiftly to over 200 countries, infecting nearly 6 million people and resulting in at least 350,000 deaths worldwide (as of 27 May 2020) [4]. An unprecedented and rapidly growing global effort is underway to develop COVID-19 vaccines and therapeutics, but at the time of this review, there are no vaccines, and only one antiviral drug (remdesivir) with modest clinical benefit has been approved under the U.S. Food and Drug Administration (FDA) Emergency Use Authorization (EUA) [5,6]. Under these circumstances, countries were forced to implement physical distancing measures to control the outbreak and, in the process, place approximately 3 billion people under lockdown.

## 2. COVID-19 Diagnostic Tests

In any infectious disease outbreak, accurate and accessible diagnostic testing must be one of the pillars of control-measure policies to understand and minimize the spread of disease. The epidemiological studies of the outbreak in China estimated the proportion of undetected COVID-19 cases to be as high as 86% [7]. As asymptomatic or mild cases could play a significant role in the transmission and spread of the SARS-CoV-2 virus [7,8], symptoms alone are not reliable diagnostic markers. There are two major types of diagnostic technologies available to address this: molecular and serological tests. Currently, much of the focus is on the SARS-CoV-2 molecular test, which can detect, with high accuracy, the virus-specific RNA molecules circulating in the host body. The gold-standard molecular test is based on reverse transcriptase polymerase chain reaction (RT-PCR) technology. However, the PCR test is not useful in distinguishing between highly infective viruses versus ones that have been neutralized by the host, and it cannot assess immunity status against SARS-CoV-2 [9]. Serologically based antibody tests can complement molecularly based tests by providing a more accurate estimate of SARS-CoV-2 incidence and by potentially detecting individuals with immunity against the disease, as these tests detect markers of the immune response.

## 3. Humoral Immune Response to SARS-COV-2

**Figure 1:**
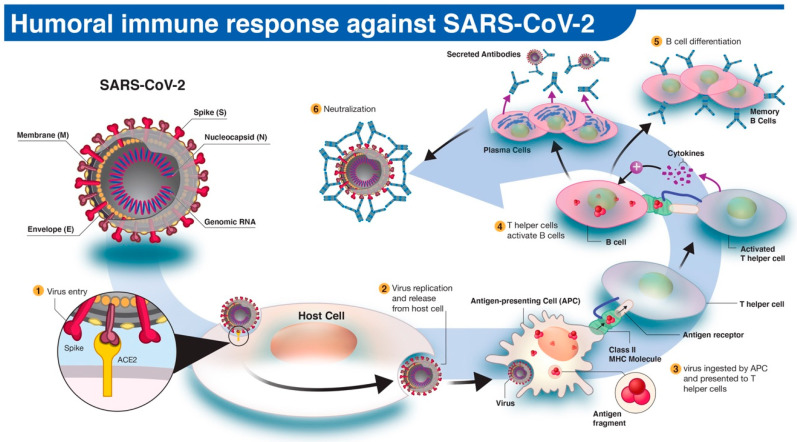
The human antibody response to SARS-CoV-2 infection. (1) The SARS-CoV-2 virus enters the host cell via interaction between viral spike (S) and host angiotensin-converting enzyme 2 (ACE2) proteins. (2,3) Following replication and release from the host cells, a subset of viruses will be engulfed and digested by antigen-presenting cells (APCs) like macrophages or dendritic cells. (4) Fragmented SARS-CoV-2 antigen(s) will be presented to T helper cells, which in turn will interact and activate B cells. (5) Activated B cells will proliferate and differentiate into plasma or memory B cells with high-affinity binding receptors for the original SARS-CoV-2 antigen. Plasma cells secrete their SARS-CoV-2-specific receptors in the form of IgM, IgG, or IgA antibodies. (6) Antibody-mediated neutralization occurs when SARS-CoV-2-specific antibodies bind to viral antigen(s) and prevent virus interaction and entry into host cells.

In humoral immune response to infection, pathogen-specific antibodies, produced by B cells, neutralize and prevent further spread of the disease. The activation and differentiation of B cells into antibody-secreting plasma B cells are triggered by a cascade of events involving virus digestion by antigen-presenting cells (e.g., dendritic cells, macrophages) and presentation of virus-specific antigens to helper T cells (Figure 1). Antibodies protect the host by binding to specific antigens (proteins) on the virus to neutralize its fusion and entry into the host cell and facilitate recognition and killing by phagocytic immune cells [10]. In humans, three types of antibodies or immunoglobulins have been the target of COVID-19 serological tests: IgM, IgG, and IgA. Although the dynamics of the immune response in COVID-19 are not fully understood, typically IgM antibodies are produced by host immune cells during the early stages of a viral infection. IgG is often the most abundant antibody in the blood and plays a more prominent role in the later stages of infection and in establishing long-term immune memory [11]. While IgM and IgG antibodies have been the leading candidates in COVID-19 serological test development, recent studies show that IgA, predominately present in the mucosal tissue, may also play a critical role in the immune response and disease progression [12].

## 4. Serological Antibody Test

Serological, or antibody, tests detect immunoglobulins produced by the host’s plasma B cells following exposure to foreign antigens. The SARS-CoV-2 genome encodes approximately 25 proteins that are required for infection and replication, including four major structural proteins: spike (S), envelope (E), membrane (M), and nucleocapsid (N) (Figure 1). The S protein plays a critical role in fusion and entry into the host cell, and it comprises an N-terminal S1 receptor-binding domain (RBD), N-terminal domain (NTD), and a C-terminal S2 subunits. The primary function of the SARS-CoV-2 N protein (NP) is binding and packing of the viral RNA genome into a helical nucleocapsid structure during viral replication [13,14]. Studies on the serum of recovered COVID-19 patients suggest that host-neutralizing antibodies primarily work against S and N proteins [15,16]. Consequently, the likelihood of predicting immunity status could increase in serological tests that target various regions of S or N proteins. Therefore, the characterization of specific SARS-CoV-2 antigen domains targeted by the humoral immune response becomes an integral part of the serological test development.

**Figure 2:**
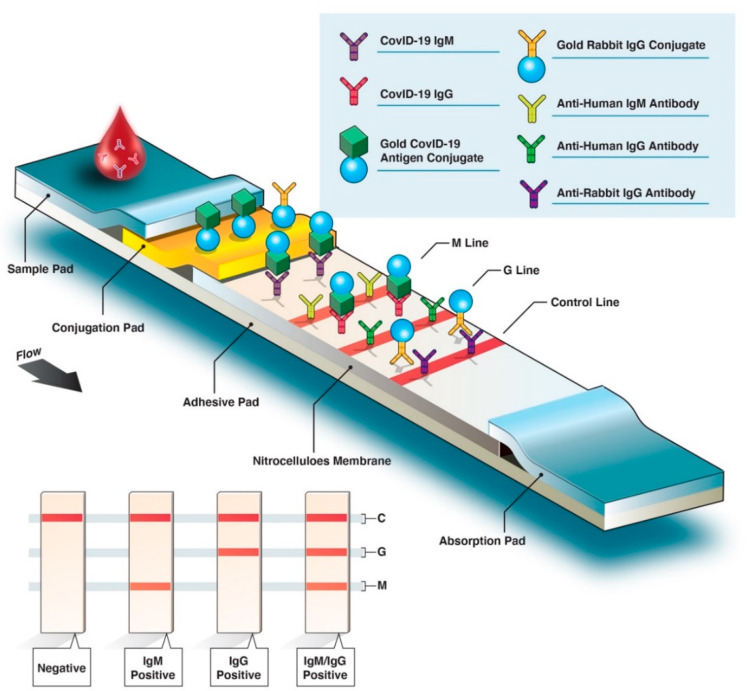
Overview of rapid diagnostic serological test. Rapid diagnostic tests (RDTs) are typically based on colorimetric lateral flow immunoassay, in which host antibodies migrate across an adhesive pad (e.g., nitrocellulose) and interact with bound virus-specific antigens and secondary antibodies (antihuman IgM/G antibodies). Conjugated SARS-CoV-2-specific antigen(s) (labeled with gold here) will bind with the corresponding host antibodies. As antibody–antigen complexes travel up the membrane, bound anti-SARS-CoV-2 IgM antibodies interact with fixed anti-IgM secondary antibodies on the M line, and anti-SARS-CoV-2 IgG antibodies interact with anti-IgG antibodies on the G line. If the blood sample does not contain SARS-CoV-2-specific antibodies, the M or G lines do not appear in the final test results; only the control (C) line will be revealed.

**Figure 3:**
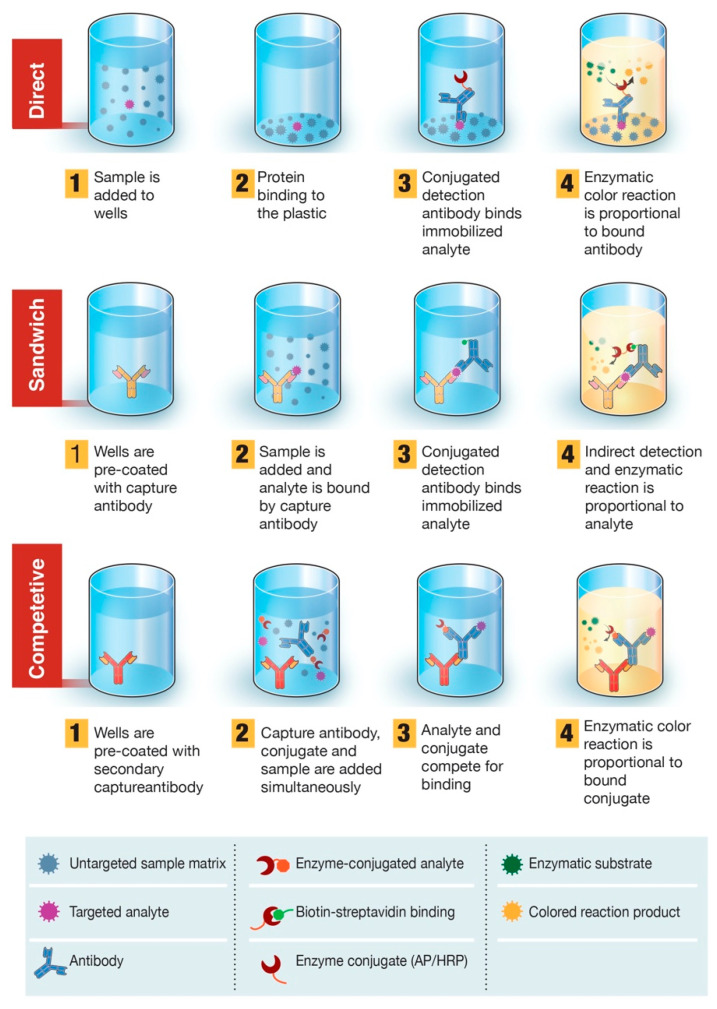
Overview of enzyme-linked immunosorbent assay (ELISA)-based diagnostic test. ELISA can be presented in different formats based on differences in antigen immobilization and antibody labeling. In direct ELISA, SARS-CoV-2 antigen(s) bound to a plastic solid phase is detected by the addition of a conjugated antibody. In sandwich ELISA, the capture antibody is attached to the plastic solid phase. Antigen(s) in the sample will bind to the capture antibody and then be detected by a second enzyme-labeled antibody. In competitive ELISA, sample SARS-CoV-2 antigen is preincubated with the primary antibody and then added to a well coated with a secondary antibody along with an enzyme-conjugated antigen that competes with the sample antigen for binding with the primary antibody. The more SARS-CoV-2 antigen in the sample, the less conjugated antigen will be bound and the lower the signal will be.

There are four major types of serological diagnostic tests: the rapid diagnostic test (RDT), enzyme-linked immunosorbent assay (ELISA), chemiluminescence immunoassay (CLIA), and neutralization assay. The neutralization assay is a lab-based test that uses live virus and cell culture methods to determine if patient antibodies can prevent viral infection in vitro. This test must be performed in laboratories with designated biosafety certificates to culture SARS-CoV-2-infected cells and has a time-to-result of 3–5 days. An RDT is a simple and rapid test based on lateral flow immunoassay (LFIA) technology, commonly found in pregnancy test kits, for example. RDT can potentially be administered as a point-of-care (POC) test or self-test. Typically, RDT test strips use a drop of blood to detect the presence of patient antibodies (IgG, IgM, or IgA) produced against a specific SARS-CoV-2 antigen (Figure 2). An RDT is simple to use with a time-to-result anywhere between 10 and 30 min. Therefore, it has the potential to be deployed in large-scale serological surveys. ELISA assay, currently the most commonly used format of the serological test, is a lab-based test with an average time-to-result of 2–5 h. ELISA typically uses a surface coated with specific viral antigen(s) to bind to and detect the corresponding patient antibodies (IgG, IgM, IgA) in blood, plasma, or serum samples. The bound antigen–antibody complex is then detected by using a second antibody and a substrate that produces a color- or fluorescent-based signal. ELISA assays can be found in different formats including direct, competitive, and, the most commonly used, sandwich or double-antigen-bridging assay (DABA) (Figure 3). CLIA technology follows a similar concept to ELISA by taking advantage of high binding affinity between the viral antigen(s) and host antibodies but uses chemical probes that yield light emission through a chemical reaction to generate a positive signal. CLIA has an average time-to-result of 1–2 h. CLIA and ELISA are both high-throughput laboratory-based assays with high level of analytical agreement [17,18].

## 5. Time Kinetics of Antibody Response in COVID-19

**Figure 4:**
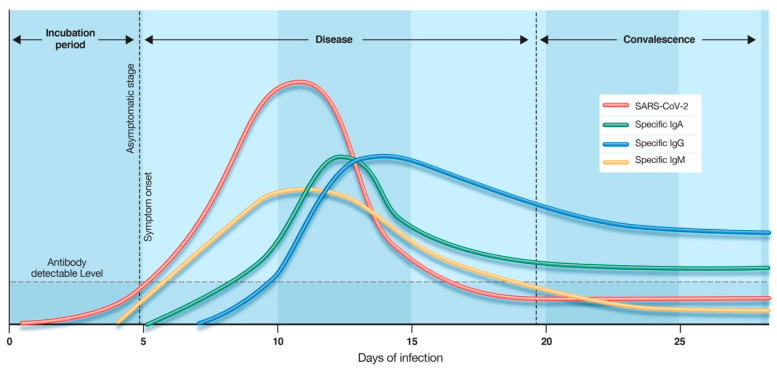
Time kinetics of antibody response in coronavirus disease 2019 (COVID-19). The illustration demonstrates the relative levels of host immunoglobulins (IgM, IgG, IgA) and SARS-CoV-2 viral load at different stages of COVID-19. Antibody-specific seroconversion occurs when the antibody reaches a detectable level in blood. Disclaimer: This graphic is for illustrative purposes only and does not represent actual levels of each antibody.

Knowledge of virus and host immune response dynamics are essential in formulating diagnostic testing and treatment strategies. Studies of COVID-19 suggest that seroconversion, when antibody levels become detectable in the blood, may take place days after the viral load has peaked [19]. Therefore, serological tests would be less effective in the early stages of COVID-19. Wolfel and colleagues further confirmed these findings by reporting IgM and IgG seroconversion in 50% of patients at 1 week after the onset of symptoms [20]. The median time for the detection of IgM and IgG in COVID-19 patients was reported to be 5 and 14 days, respectively [21]. Yu and colleagues detected the seroconversion of IgA on day 2 and IgM/IgG on day 5 after onset of symptoms. Furthermore, the study reported that 100% of cases had detectable levels of IgA, IgM, and IgG on day 32 after onset of symptoms [12]. Their findings also revealed IgM and IgG levels to be significantly higher in severe COVID-19 cases than in patients with mild or moderate disease [12], suggesting that serological tests require high sensitivity to detect lower levels of antibodies in mild cases. Studies on the persistence of antibodies in blood suggest that high levels of IgG are detectable for at least 49 days after the onset of symptoms, while IgM levels declined rapidly on day 35 postinfection [22]. The diagram in Figure 4 depicts the timelines and peak levels for SARS-CoV-2 viral load relative to blood IgM, IgG, and IgA antibodies. Improved understanding of humoral antibody response time kinetics in COVID-19 is crucial to the correct application of serological tests.

## 6. Serological Test Performance

The urgent need for the development of serological diagnostic tests in response to the COVID-19 outbreak has compelled regulatory bodies to implement emergency use authorization programs to expedite the commercialization process of these tests. In light of this, independent and robust post-market evaluations of COVID-19 serological tests are needed to confirm manufacturers’ performance claims. The basic measures of quantifying diagnostic test performance are sensitivity and specificity. Sensitivity is the ability of a test to detect the disease agent or the host’s response to the disease (i.e., antibodies) when it is truly present, whereas specificity is the ability of a test to correctly return a negative result when disease or host response is absent [23].

We conducted a systematic review of independent studies that assessed the performance of currently available SARS-CoV-2 serological tests. We included studies that reported sensitivity and specificity, stage of disease (early, intermediate, or late), the test format (CLIA, ELISA, RDT), and antibody target (IgA, IgG, IgM, or IgG + IgM) [24,25]. If available, the SARS-CoV-2 antigens used for antibody detection was recorded. The studies that did not specify the disease stage of test samples were grouped under the “overall” category and assessed separately. In total, we reviewed performance data on 5 serological CLIA tests, 15 serological ELISA tests, and 42 serological RDTs currently on the market (see Supplementary Materials).

**Figure 5:**
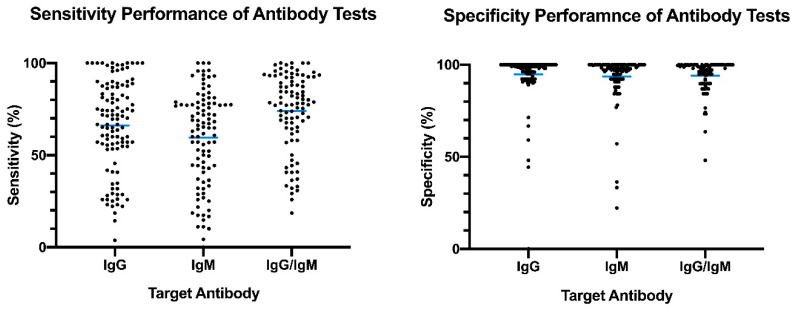
Overview of reported sensitivity and specificity of COVID-19 serological tests. Reported performance data from categories were plotted based on target antibody (IgG, IgM, IgG/IgM) to provide an overview of variability distribution in marketed diagnostic tests. Blue bars represent the mean values.

**Figure 6:**
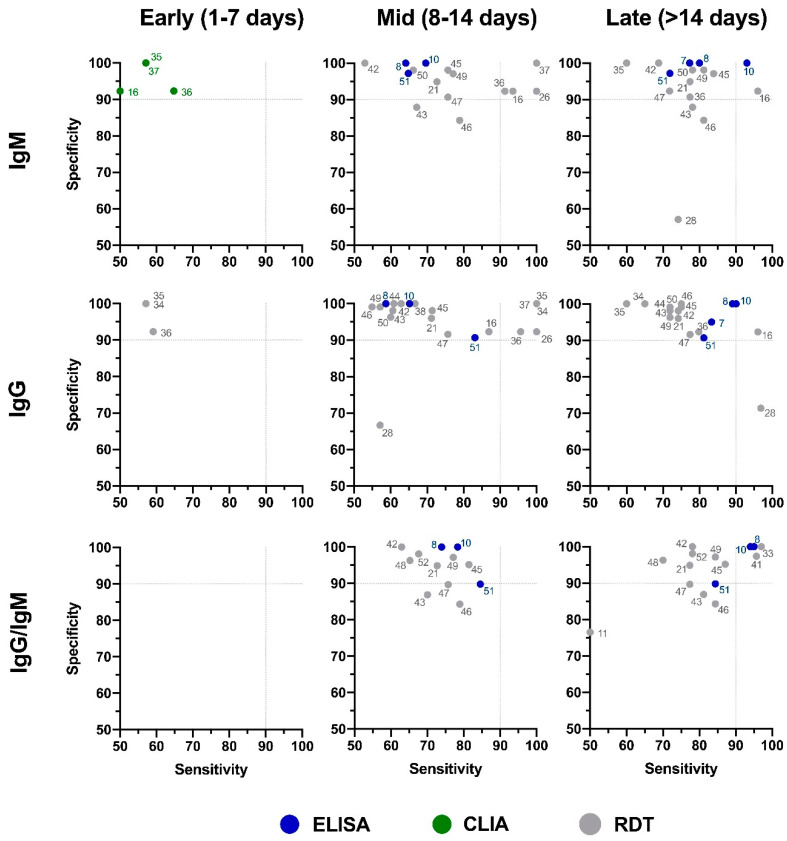
Independent evaluation of COVID-19 serological tests at various stages of the disease. XY plots of reported sensitivity and specificity in detection of IgM, IgG, or IgG/IgM antibodies were generated at different stages of the disease, including early (1–7 days after symptom onset), intermediate (7–14 days after symptom onset), and late (>14 days after symptom onset). ELISA: enzyme-linked immunosorbent assay; CLIA: chemiluminescence immunoassay; RDT: rapid diagnostic test (lateral flow immunoassay). Dotted lines represent 90% sensitivity and specificity levels. Refer to Figure 7 for test names and manufacturers associated with number shown here.

**Figure 7:**
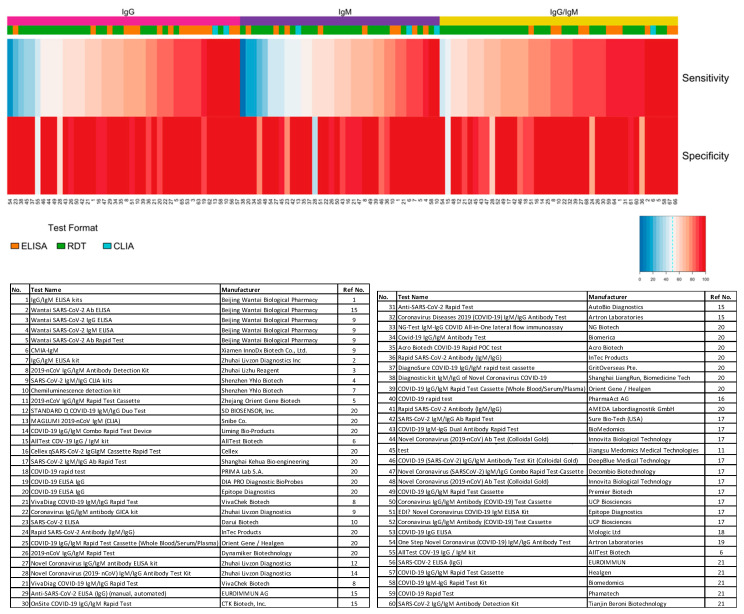
Independent evaluation of SARS-CoV-2 serological test overall performance. Sensitivity and specificity data from studies that did not specify the COVID-19 stage (“overall” group) are represented in a heatmap. The heatmap is ordered according to the antibody target (IgG, IgM, and IgG/IgM) followed by sensitivity and specificity values. All analyses were conducted using software R version 3.6.3. Heatmaps were generated using the gplots and RColorBrewer packages. ELISA: enzyme-linked immunosorbent assay; CLIA: chemiluminescence immunoassay; RDT: rapid diagnostic test.

The distribution plot of the data shows a higher degree of variability in test sensitivity values compared to specificity (Figure 5). This level of variability further emphasizes the need for independent evaluations of serological tests on the market. The sensitivity/specificity plots highlight tests at various stages of COVID-19 and confirm the expectation that serological tests are more effective in later stages of the disease when higher IgG and IgM levels are present in the blood (Figure 6). The heatmap of tests in the “overall” category ranks the highest-performing test in each target antibody category based on sensitivity, followed by specificity (Figure 7). Top-performing COVID-19 serological tests (>95% sensitivity and specificity) from the XY plots and heatmap are summarized in Table 1.

## 7. Seroprevalence of SARS-COV-2-Specific Antibodies

In light of policies to ease the lockdown and reopen the economy, large-scale seroprevalence studies to screen for immunity status are being implemented in several jurisdictions. Critics point to gaps in our understanding of immune response to COVID-19 infection, including the ability of serological tests to detect neutralizing antibodies and the capacity of the immune system to provide long-term immunity against SARS-CoV-2. However, some argue that in the context of a global viral outbreak with a relatively high mortality rate, inaction due to uncertainty can have negative consequences compared to the harm caused by false-positive and false-negative serological test results [31]. Several jurisdictions have initiated seroprevalence studies to provide a more accurate estimate of cases with positive SARS-CoV-2-specific antibodies, irrespective of disease symptoms. In Los Angeles County, the prevalence of SARS-CoV-2 antibodies in the community was estimated to be 4.65%, equivalent to 367,000 adults, which was substantially greater than the 8430 confirmed cases in the same county at the time of the study [32]. In New York City, 19.9% of the population has been estimated to have SARS-CoV-2 antibodies, compared to 2.1% confirmed cases as of 2 May 2020 [33]. Similar studies from Germany, the U.K., Singapore, and China show significantly higher estimates of positive SARS-CoV-2 antibody cases compared to symptomatic cases confirmed by molecular tests [34]. As undetected cases with mild or no symptoms can transmit the virus, it is not surprising that countries (e.g., South Korea, Germany, and Singapore) with large-scale and well-organized testing programs, combined with extensive isolation and contact tracing for infected individuals, have had some success in minimizing COVID-19-related death in their populations [35].

## 8. Concluding Remarks

As serological tests are in high demand, in part due to an increase in large-scale seroprevalence studies, it is imperative for national and regional governments to continue coordinated efforts to independently validate serological test performance and partner with industry to scale up manufacturing and production capacity. Existing emergency authorization programs, intended to accelerate the manufacturing of diagnostic tests, must also be accompanied by clear and informed guidelines on preferred and minimally acceptable profiles of COVID-19 serological tests designed for specific indications.

Despite the unprecedented response to the outbreak, major gaps remain in our understanding of the interaction between SARS-CoV-2 and the immune system, which can negatively impact serological testing utilization. Coordinated research efforts are urgently needed to investigate some of the key gaps in our knowledge, including:

1. Which serological tests can identify SARS-CoV-2-neutralizing antibodies?
2. Is there cross-reactivity between neutralizing antibodies and other coronaviruses?
3. Which SARS-CoV-2 antigens are optimal for the detection of neutralizing antibodies?
4. What is the correlation between SARS-CoV-2-specific antibodies and protective immunity status?
5. How long does protective immunity last in recovered patients? Are individuals susceptible to reinfection with SARS-CoV-2?
6. Is humoral antibody response the best indicator for protective immunity, or are there other immune-cell-based mechanisms?

In the context of the COVID-19 outbreak and the execution of return-to-work policies, failing to take advantage of available diagnostic tools due to uncertainty can have profound consequences. Medical professionals frequently rely on imperfect evidence with the possibility of false positives and false negatives. It is, however, important to clearly understand the limits and potential of serological tests to make informed decisions based on risk and benefit assessment in each specific situation. In the words of Tedros Adhanom Ghebreyesus, Director-General of the World Health Organization, “Countries cannot fight this pandemic blindfolded. Countries should know where the cases are.”

## Figures and Tables

**Table 1:** Top-performing COVID-19 serological tests based on independent evaluations of sensitivity/specificity.

No.	Test Name [ref]	Manufacturer	Format	Target Antibody	Disease Stage	Sensitivity (%)	Specificity (%)	Antigen
2	Wantai SARS-CoV-2 Ab ELISA [26]	Beijing Wantai Biological Pharmacy	ELISA	IgG/IgM/IgA	Overall	95.4	100	RBD
8	2019-nCoV IgG/IgM Antibody Detection Kit [27]	Zhuhai Lizhu Reagent	ELISA	IgG/IgM	Late (>14 days)	95	100	NP
6	CMIA-Ab [28]	Xiamen InnoDx Biotech Co., Ltd.	CLIA	IgG/IgM	Overall	96.2	99.3	RBD
10	Chemiluminescen. detection kit [29]	Shenzhen Yhlo Biotech	CLIA	IgM IgG	Overall	100 100	98.5 99	n/a
13	MAGLUMI 2019-nCoV IgG [25]	Snibe Co.	CLIA	IgG	Overall	98.8	95.1	n/a
61	Architect SARS-CoV-2 IgG Assay [30]	Abbott	CLIA	IgG	Late (>14 days)	97.2	100	NP
5	Wantai SARS-CoV-2 Ab Rapid Test [28]	Beijing Wantai Biological Pharmacy	RDT	IgG/IgM/IgA	Overall	97.5	95.2	RBD
33	NG-Test IgM-IgG COVID All-in-One Lateral Flow Immunoassay [25]	NG Biotech	RDT	IgG/IgM	Late (>14 days)	97	100	n/a
34	Covid-19 IgG/IgM Antibody Test [25]	Biomerica	RDT	IgG	Mid. (8–14 days)	100	100	n/a
35	Acro Biotech COVID-19 Rapid POC Test [25]	Acro Biotech	RDT	IgG	Mid. (8–14 days)	100	100	n/a
37	DiagnoSure COVID-19 IgG/IgM Rapid Test Cassette [25]	GritOverseas Pte.	RDT	IgG IgM	Mid. (8–14 days)	100 100	100 100	n/a
41	Rapid SARS-CoV-2 Antibody (IgM/IgG) [25]	AMEDA Labordiagnostik GmbH	RDT	IgG/IgM	Late (>14 days)	95.7	97.4	n/a
57	COVID-19 IgG/IgM Rapid Test Cassette [24]	Healgen	RDT	IgM IgG	Overall	100 96.7	100 97.5	n/a

## Supplementary Materials

The following are available online at https://www.mdpi.com/2075-4418/10/7/453/s1.

## Abbreviations

COVID-19	Coronavirus Disease 2019
SARS	Severe Acute Respiratory Syndrome
SARS-CoV	Severe Acute Respiratory Syndrome Coronavirus
SARS-CoV-2	Severe Acute Respiratory Syndrome Coronavirus 2
MERS	Middle East Respiratory Syndrome
BAL	Bronchoalveolar Lavage
EUA	Emergency Use Authorization
RT-PCR	Reverse Transcriptase Polymerase Chain Reaction
S	SARS-CoV-2 Spike Protein
E	SARS-CoV-2 Envelope Protein
M	SARS-CoV-2 Membrane Protein
NP	SARS-CoV-2 Nucleocapsid Protein
RBD	Receptor-Binding Domain
ELISA	Enzyme-Linked Immunosorbent Assay
RDT	Rapid Diagnostic Test
CLIA	Chemiluminescence Immunoassay
LFIA	Lateral Flow Immunoassay
PoC	Point of Care
DABA	Double-Antigen-Bridging Assay
